# Effect of pore structure in bismuth metal–organic framework nanorod derivatives on adsorption and organic pollutant degradation

**DOI:** 10.1039/d4ra05657d

**Published:** 2024-09-30

**Authors:** Vy Anh Tran, Tran Thanh Sang, Nguyen Anh Thu, Vien Vo, Van Thuan Le, Van Dat Doan, Thu-Thao Thi Vo, Nguyen Duy Dat

**Affiliations:** a Department of Material Science, Institute of Applied Technology and Sustainable Development, Nguyen Tat Thanh University Ho Chi Minh City 700000 Vietnam tavy@ntt.edu.vn; b Faculty of Chemical & Food Technology, University of Technology and Education Thu Duc Ho Chi Minh City 700000 Vietnam datnd@hcmute.edu.vn; c Faculty of Natural Sciences, Quy Nhon University 170 An Duong Vuong Quy Nhon Binh Dinh 55000 Viet Nam; d Center for Advanced Chemistry, Institute of Research and Development, Duy Tan University 03 Quang Trung Da Nang 550000 Vietnam; e The Faculty of Chemical Engineering, Industrial University of Ho Chi Minh City Ho Chi Minh City 700000 Vietnam; f Department of Food Science and Biotechnology, Gachon University 1342 Seongnamdaero, Sujeong-gu Seongnam-si 13120 Republic of Korea vothuthaobd@gmail.com

## Abstract

This study explores the synthesis, characterization, and photocatalytic properties of bismuth metal–organic framework (Bi-MOF) nanorods and their derivatives such as Ag/Bi-MOF and Ag/Bi_2_O_3_. Bi-MOF nanorods exhibit significant photocatalytic activity under visible light, with the addition of silver (Ag) enhancing electron–hole pair separation and reducing their recombination. This leads to improved photocatalytic performance, particularly in the degradation of organic pollutants such as Rhodamine B (RhB) and Methylene Blue (MB). The results show that Bi-MOF and its derivatives demonstrate excellent chemical stability and high performance in photocatalytic applications, even when subjected to high temperatures and tested across a wide pH range. The large surface area and microporous structure facilitate selective adsorption of small organic molecules like MB. The pores and large surface area not only provide numerous active sites but also enhance the interaction between reactants and the catalyst surface, improving photocatalytic efficiency. Bi-MOF and its derivatives perform optimally across a broad pH range, from acidic to alkaline environments, where strong oxidizing hydroxyl radicals (·OH) are easily formed, aiding in the effective degradation of organic compounds. The study also shows that Bi-MOF and its derivatives can be reused multiple times without significant loss in performance. This research contributes to the development of advanced materials for environmental remediation, highlighting the potential of Bi-MOF-based nanocomposites in practical applications.

## Abstract 2

This study explores the synthesis, characterization, and photocatalytic properties of bismuth metal–organic framework (Bi-MOF) nanorods and their derivatives such as Ag/Bi-MOF and Ag/Bi_2_O_3_.

## Graphical abstract

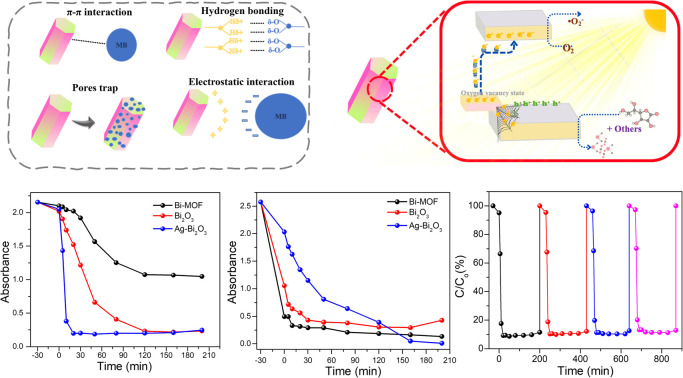

## 1. Introduction

Metal–organic frameworks (MOFs) are a diverse class of materials known for their large surface area, adjustable porosity, and multifunctional capabilities.^1–3^ Bismuth-based MOFs have received significant interest because of their distinct properties and potential uses in various domains, including catalysis, sensing, and environmental cleanup.^4–7^ Bismuth MOF nanorods and their derivatives, such as Ag/Bi_2_O_3_, are an exciting research area with substantial potential across many fields.^8–11^ Their characteristics, including high surface area, photocatalytic activity, antibacterial properties, chemical stability, electrochemical performance, and optical properties, make them highly adaptable for numerous applications.^12–15^ Ongoing research and development are anticipated to further improve their properties and broaden their practical applications, contributing to progress in environmental remediation, healthcare, energy storage, and sensing technologies.^16–20^

Bismuth MOF nanorods and their derivatives, such as Ag/Bi_2_O_3_, display a variety of exceptional properties that make them highly desirable for diverse applications. These materials possess a high surface area and porosity, offering numerous active sites for catalytic reactions and facilitating molecule adsorption, which is beneficial for applications like gas storage, separation, and catalysis. Additionally, both bismuth MOF nanorods and Ag/Bi_2_O_3_ show significant photocatalytic activity under visible light.^21–23^ The inclusion of silver improves charge separation efficiency and reduces electron–hole recombination, thus enhancing photocatalytic performance.^24,25^ This makes them suitable for environmental remediation applications, such as degrading organic pollutants and purifying water. Furthermore, bismuth MOF nanorods and their derivatives maintain excellent chemical stability in various environments, ensuring their structural integrity and functionality over extended periods, which is essential for long-term practical use.^26^ The unique structure of bismuth MOF nanorods, combined with the presence of silver in Ag/Bi_2_O_3_, boosts their electrochemical properties, making them ideal for energy storage devices like batteries and supercapacitors,^27,28^ where high surface area and conductivity are critical for efficient performance.^29,30^ These materials possess distinctive optical properties, including visible light absorption and photoluminescence, which are advantageous for applications in sensing and optoelectronics, where the ability to detect and interact with light is crucial.^31,32^

The robust coordination bonds between the bismuth ions and organic linkers contribute to the high thermal stability of these materials. Studies have shown that certain Bi-MOFs can retain their crystalline structure and porosity at temperatures exceeding 500 °C. This thermal stability ensures that the materials do not degrade or lose their structural integrity, which is crucial for maintaining consistent catalytic activity under prolonged high-temperature conditions.^33,34^ Bi-MOFs and their derivatives also demonstrate excellent chemical stability across a broad pH range.^35,36^ This stability is attributed to the strong metal–organic coordination bonds that are resistant to hydrolysis and chemical attack. Bi-MOFs have been shown to maintain their structural integrity and functional properties in highly acidic (pH ∼1) and highly alkaline (pH ∼13) environments. This wide pH stability is essential for catalytic applications in diverse chemical environments, including wastewater treatment and industrial catalysis, where pH conditions can vary significantly.^37–39^ The reusability of Bi-MOFs and their derivatives is a critical factor for practical applications in adsorption and catalysis. These materials have shown minimal loss in performance after multiple cycles of use. The stability of the framework and the active sites ensures that the catalytic efficiency and adsorption capacity remain high even after repeated use.^40,41^

This study focused on synthesized and characterized bismuth metal–organic framework (Bi-MOF) nanorods and their derivatives, such as Bi_2_O_3_, and Ag/Bi_2_O_3_, which showed significant photocatalytic activity under visible light. The addition of silver improved charge separation efficiency and reduced electron–hole recombination, leading to enhanced degradation of organic pollutants like Rhodamine B and Methylene Blue. Bi-MOFs and their derivatives were investigated the chemical stability and high efficiency in photocatalytic applications, even at high temperatures and across a wide pH range. Their large surface area and microporous structure facilitated the selective adsorption of small molecules like MB, significantly impacting the adsorption process. Additionally, these materials can be reused multiple times without significant performance loss, highlighting their potential for sustainable and efficient environmental remediation applications.

## 2. Methods & experiment

### 2.1. Chemical materials

1,4-Benzoic acid, bismuth(iii) nitrate pentahydrate, silver nitrate, ammonium chloride, *N*,*N*-dimethylformamide, rhodamine B, methylene blue, sulfuric acid (0.1 N), and sodium hydroxide (0.1 N) were sourced from Sigma-Aldrich company. Ethanol and HPLC-grade water were used without additional purification. All other chemicals were of the highest commercially available grade and used as supplied. Glassware was cleaned with a solution of HNO_3_ (3 : 1) and subsequently rinsed multiple times with deionized (DI) water.

### 2.2. Synthesis of pristine bismuth-MOF nanorod (Bi-MOF) and bismuth oxide (Bi_2_O_3_)

6 mmol Bi(NO_3_)_3_·5H_2_O (2.91 g) and 9 mmol H_2_BDC (1.494 g) are dissolved in 60 mL of DMF solution, then the mixture is stirred at 500 rpm for 30 minutes until completely dissolved and transparent. The solution is then transferred to a Teflon-lined autoclave and heated at 100 °C for 72 hours. Afterward, the mixture is cooled to room temperature and centrifuged at 3000 rpm for 10 minutes, repeated three times. The mixture is then washed with DMF, and the catalyst is separated. It is centrifuged again at 6000 rpm for 6 minutes with 40 mL of ethanol, repeated three times, to remove any remaining substances. The final mixture is dried at 120 °C for 12 hours to obtain Bi-MOF. Subsequently, 50 mg of the Bi-MOF nanorods are calcined in a tube furnace at 450 °C for 3.5 hours. The product is then cooled to room temperature to yield Bi_2_O_3_ material.^42,43^

### 2.3. Synthesis of Ag/Bi-MOF and Ag–Bi_2_O_3_ nanorod

Weigh 2.91 grams of Bi(NO_3_)_3_·5H_2_O (6 mmol) and 0.51 grams of AgNO_3_ (3 mmol) in a molar ratio of 1 : 2 for AgNO3 : Bi(NO_3_)_3_·5H_2_O. Dissolve these in 60 mL of DMF and stir for 30 minutes until the solution is completely clear. Transfer the clear solution into a Teflon-lined autoclave and seal it tightly. Heat the autoclave at 100 °C for 72 hours, then allow the mixture to cool to room temperature. To remove any remaining reactants, centrifuge the mixture at 3000 rpm for 10 minutes, repeating this process three times. Wash the mixture with DMF, isolate the catalyst, and centrifuge again at 3000 rpm for 10 minutes with approximately 40 mL of ethanol, repeating this process three times. Dry the final mixture obtained after the washing process at 120 °C for 12 hours to produce the Ag/Bi-MOF product. To obtain Ag/Bi_2_O_3_, calcine 500 mg of the Ag/Bi-MOF nanorods in a tube furnace at 450 °C for 3.5 hours, then cool the product to room temperature.^21,44^

### 2.4. Adsorption experiments

Batch experiments were carried out to assess the adsorption efficiency of Bi-MOF, Bi_2_O_3_, and Ag/Bi_2_O_3_ at 25 °C. The experiments involved dissolving specified amounts of RhB and MB in deionized (DI) water. During the first cycle of the adsorption process, 50 mg of Bi-MOF/Bi_2_O_3_/Ag/Bi_2_O_3_ was suspended in 100 mL of solution containing 30 ppm RhB and 50 ppm MB at pH 7. The mixture was sonicated for 1 minute and then transferred to a reactor, where it was stirred at 400 rpm on a magnetic stirrer at 25 °C for 30 minutes. The reactor's temperature was kept constant through water circulation in an outer jacket surrounding the reactor. The adsorbent was separated from the solution using centrifugation. To remove RhB and MB from the Bi-MOF and its modifications, the adsorbent was immersed in abundant ethanol solvent under constant stirring. Subsequently, the Bi-MOF/Bi_2_O_3_/Ag/Bi_2_O_3_ was dried in a vacuum oven for subsequent experiments.

At the end of the adsorption cycles, the absorbance of residual RhB and MB in the supernatant was measured at 662 nm and 553 nm using a UV/vis spectrophotometer. The adsorption capacity of Bi-MOF/Bi_2_O_3_/Ag/Bi_2_O_3_ for RhB/MB was calculated by the following equation:1
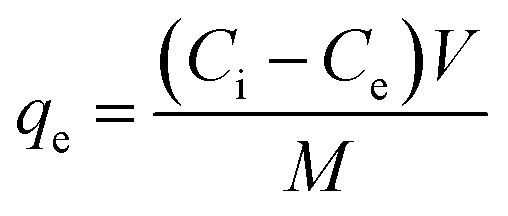
where *q*_e_ (mg g^−1^) is the equilibrium adsorption capacity of Bi-MOF/Bi_2_O_3_/Ag/Bi_2_O_3_ for RhB/MB; *C*_i_ and *C*_e_ are the initial and equilibrium concentration of RhB/MB (mg L^−1^), respectively. *V* is the volume of RhB/MB in Liter. *M* is the adsorbent dosage (g). The adsorption percentage (percentage ratio of the removed RhB/MB to the existing RhB/MB in the stock solution) is defined as follows:2
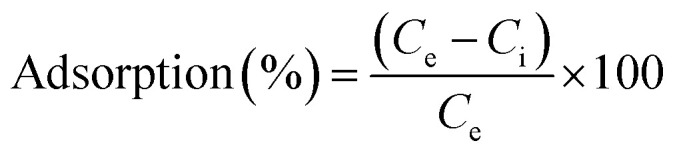


### 2.5. Photocatalytic experiments

The photocatalytic performance of the sample was assessed by measuring the photodegradation percentage of RhB/MB in an aqueous solution under visible light exposure. A 250 W high-pressure mercury lamp emitting at 365 nm served as the visible light source. Typically, 50 mg of the Bi-MOF/Bi_2_O_3_/Ag/Bi_2_O_3_ photocatalyst was added to a photoreactor containing 100 mL of RhB/MB solution. The photoreactor's temperature was maintained at 25 °C using circulating cooling water in an outer jacket around the reactor. Before light exposure, the dye solution was stirred in the dark for 30 minutes to achieve adsorption–desorption equilibrium. The dye solution was then exposed to UV light. At specified time intervals, 3 mL aliquots were taken from the photoreactor and centrifuged to separate the photocatalysts. The supernatant was used to monitor the photodegradation percentage of RhB/MB dye. The absorbance of the sampled RhB/MB dye was recorded every 10 minutes at their maximum absorption wavelengths of 662 nm and 553 nm, respectively.

### 2.6. Physicochemical properties of MOF nanomaterials

#### 2.6.1 Scanning electron microscopy (SEM)

A Hitachi S-4700 FE-SEM (Japan) was employed to examine the surface morphology of the MOF samples at an accelerating voltage of 15 kV. The samples were applied to cover glasses (22 × 22 mm), which were then attached to polished aluminum stubs using carbon tape after drying. To enhance the accuracy of size measurements in SEM analysis, the samples were not coated with palladium or gold. Before being applied to the glass to reduce nanoparticle aggregation, all sample solutions were treated in an ultrasonic bath for 30 seconds and then mixed with a vortex mixer for 20 seconds.

#### 2.6.2 Fourier transform infrared spectra (FTIR)

After freeze-drying the powder sample, a small amount is placed between two support plates without any hygroscopic material. The FTIR spectra were recorded using a Vertex 70 FTIR spectrometer (Bruker, USA).

#### 2.6.3 X-ray diffraction

The XRD patterns of the nanoparticles were generated using an X-ray automated diffractometer (Rigaku Rint 2200 Series, Rigaku, Japan) operating in continuous-scanning 2*θ* mode (40 kV, 30 mA). The patterns were created using monochromatized Cu Kα1 radiation with a wavelength of 1.5406 Å.

#### 2.6.4 Nitrogen physisorption isotherms

Nitrogen physisorption isotherms were measured at −196 °C using a volumetric apparatus. Before testing, samples were outgassed in a vacuum at room temperature for a minimum of 30 hours. Surface areas were calculated using the Brunauer–Emmett–Teller (BET) method, while the Barrett–Joyner–Halenda (BJH) method was employed to determine the mesopore size distribution.

## 3. Results & discussion

### 3.1. Preparation and characterization of as-prepared samples

The chemical structure of Bi-MOF is characterized by the unique coordination between bismuth ions and organic ligands, forming a three-dimensional porous network. Bismuth ions serve as the main metal centers in the framework. These ions tend to form strong coordination bonds due to their relatively large ionic radius and stereochemically active lone electron pair. The organic components in Bi-MOF consist of carboxylate groups that connect the metal ions. These organic molecules act as bridges, linking the bismuth ions through carboxylate groups, and facilitating the construction of the framework. Bi-MOF can be modified or functionalized after synthesis to enhance its properties. This includes incorporating functional groups on the organic ligands or within the pores.

#### 3.1.1 FTIR analysis

**Fig. 1:**
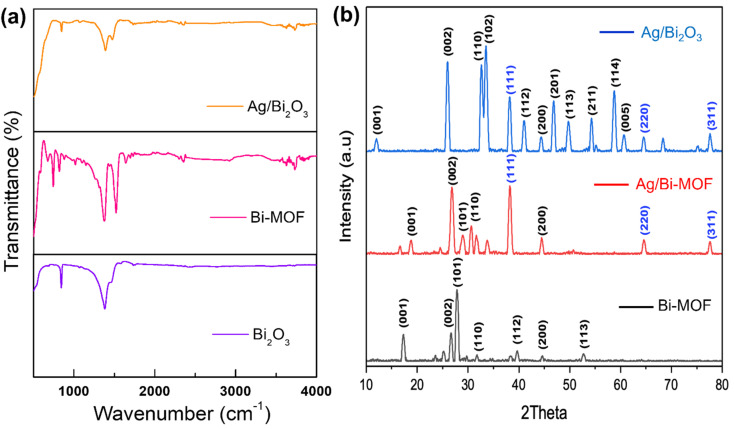
(a) FTIR spectra of Bi-MOF, Bi_2_O_3_, and Ag/Bi_2_O_3_, (b) XRD spectra of Bi-MOF, Ag/Bi-MOF, and Ag/Bi_2_O_3_.

The FTIR spectrum of Bi-MOF nanorods reveals several key features indicative of the material's structure and composition (Fig. 1a). A broad peak typically observed in the range of 3511 cm^−1^ corresponds to the O–H stretching vibrations from hydroxyl groups. In the 2930 cm^−1^ range, peaks are attributed to the C–H stretching vibrations of carboxylates and/or aromatic rings. A peak in the 1653 cm^−1^ range indicates the presence of carbonyl groups (C=O), common in carboxylate ligands used in Bi-MOF. Peaks in the 1518 cm^−1^ range are associated with the C=C stretching vibrations of aromatic rings. The 1376 cm^−1^ range features peaks due to the stretching vibrations of C–O bonds in carboxylate groups and Bi–O bonds, indicating the coordination of bismuth ions with oxygen atoms from the ligands. The region of 747–823 cm^−1^, contains a series of peaks corresponding to bending and stretching vibrations of various bonds unique to the specific Bi-MOF structure.^45^ The FTIR spectrum of Bi_2_O_3_ formed from the calcination of Bismuth MOF nanorods is primarily characterized by the strong Bi–O stretching vibrations in the 500 cm^−1^ range. The absence of peaks related to organic ligands confirms the decomposition of the MOF structure and the formation of pure bismuth oxide. Additionally, the results retained the fundamental peaks of Bi-MOF at 1463 and 1399 cm^−1^. Analyzing this spectrum and comparing it with standard spectra of Bi_2_O_3_ helps verify the successful conversion and purity of the resulting oxide.^46^ The FTIR spectrum of Ag-doped Bi_2_O_3_ formed from the calcination of bismuth MOF nanorods is characterized by strong Bi–O stretching vibrations in the 500 cm^−1^ range. The introduction of Ag can cause additional peaks or shifts in the spectrum due to the formation of Ag–O bonds. The absence of peaks related to organic ligands confirms the decomposition of the MOF structure and the formation of the doped bismuth oxide. The FTIR spectra provide detailed insights into the functional groups and bonding characteristics of Bi-MOF, Bi_2_O_3_, and Ag/Bi_2_O_3_. The presence of characteristic peaks for Bi–O bonds confirms the formation of bismuth oxide structures, while the differences in peak intensity and position indicate the successful incorporation of silver into the Bi_2_O_3_ matrix.

#### 3.1.2 XRD analysis

The XRD spectra display the diffraction patterns of Bi-MOF, Ag/Bi-MOF, and Ag/Bi_2_O_3_, highlighting their distinct crystallographic phases (Fig. 1b). The Bi-MOF spectrum shows distinct peaks corresponding to various crystallographic planes. The peak at 2*θ* ≈ 27.2° corresponds to the (002) plane, which is a characteristic reflection of Bi-MOF. Additional peaks at 2*θ* ≈ 28.5°, 32.0°, and 39.8°, 44.8°, and 53.0° represent the (101), (110), (112), (200), and (113) planes, respectively.^21,47^ These peaks indicate the presence of a well-defined crystalline structure typical for Bi-MOF. The Ag/Bi-MOF spectrum exhibits peaks at similar positions to those of Bi-MOF but with additional peaks indicating the presence of Ag. The peak at 2*θ* ≈ 38.2° corresponds to the (111) plane, which is characteristic of the face-centered cubic (FCC) structure of Ag.^48^ The intensity of the peaks suggests that the incorporation of Ag into the Bi-MOF framework leads to slight modifications in the crystallographic structure, evident by the shift and additional peaks at 2*θ* ≈ 64.8° and 77.8° for the (220) and (311) planes.^49,50^ The Ag/Bi_2_O_3_ spectrum shows a complex pattern with numerous peaks indicating the formation of a new crystalline phase. Peaks correspond to the planes of Bi_2_O_3_ and Ag, respectively. The doping of Ag into Bi-MOF leads to noticeable changes in the crystalline structure, while the formation of Ag/Bi_2_O_3_ results in a new and more complex crystalline phase. These structural differences can significantly impact the material's properties and potential applications in various fields such as catalysis and adsorption.

#### 3.1.3 BET analysis

**Fig. 2:**
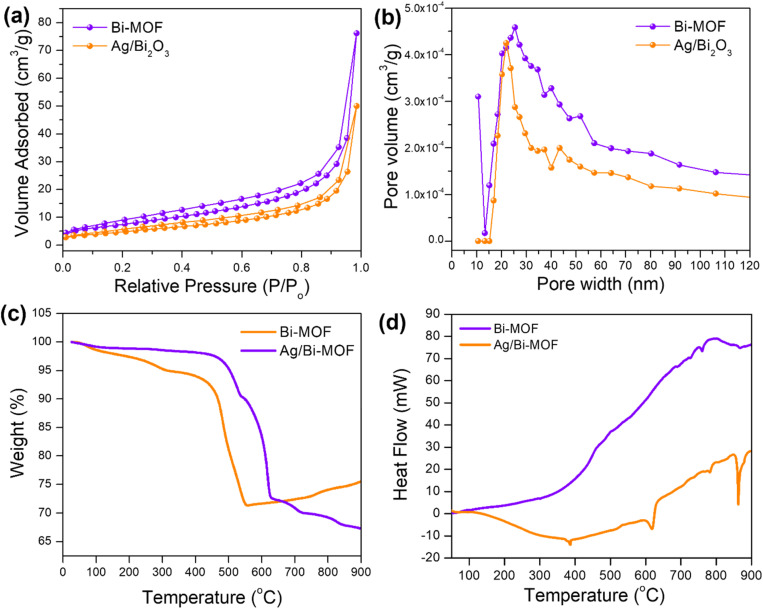
(a) Nitrogen adsorption–desorption isotherm of Bi-MOF, Ag/Bi_2_O_3_, (b) pore size distribution of Bi-MOF, Ag/Bi_2_O_3_, (c) thermogravimetric analysis (TGA) and (d) differential scanning calorimetry (DSC) of Bi-MOF, Ag/Bi-MOF in the atmosphere.

Brunauer–Emmett–Teller (BET) analysis shows the nitrogen adsorption–desorption isotherms and Barrett–Joyner–Halenda (BJH) method was used for pore size distribution of Bi-MOF and Ag/Bi_2_O_3_, providing insights into their porous structures (Fig. 2a). The adsorption/desorption isotherms are close to type II isotherms. At the initial part of the adsorption isotherm, the adsorption capacity increases sharply, which is a clear indication of the abundant nanopores.^51–53^ The overall higher adsorption capacity indicates a larger surface area and higher pore volume compared to Ag/Bi_2_O_3_. Bi-MOF has a higher nitrogen adsorption capacity (35.09 m^2^ g^−1^), indicating a larger specific surface area and higher pore volume than Ag/Bi_2_O_3_ (20.69 m^2^ g^−1^). This is due to the coverage of Ag nanoparticles on the bismuth nanorods during the formation of Ag/Bi_2_O_3_. This suggests that Bi-MOF provides more active sites for adsorption processes.^54^ The pore size distribution curve for Bi-MOF shows a peak at smaller pore widths (25 nm), contributing to its higher pore volume. The pore size distribution for Ag/Bi_2_O_3_ shows a slight decrease (22 nm), indicating fewer mesopores and a smaller pore volume.^7,55^ This distribution is narrower compared to Bi-MOF. The presence of mesopores is evident, but the volume and surface area are reduced compared to Bi-MOF. The BET analysis reveals that Bi-MOF possesses a higher surface area and pore volume compared to Ag/Bi_2_O_3_, attributed to its broader and more significant mesoporous structure. These characteristics make Bi-MOF more suitable for applications requiring high adsorption capacities and extensive surface interactions. On the other hand, Ag/Bi_2_O_3_, with its more uniform and fewer mesopores, might be preferred in applications where specific and controlled interactions are crucial (Fig. 2b).

#### 3.1.4 TGA and DSC analysis

The TGA-DSC analysis for Bi-MOF and Ag/Bi-MOF provides their thermal behavior, decomposition, and stability (Fig. 2c and d). The TGA curve for Bi-MOF typically shows several weight loss steps corresponding to different thermal events. Initial weight loss at temperatures below 100 °C is likely due to the evaporation of adsorbed solvents. A significant weight loss step around 500 °C indicates the decomposition of the organic ligands in the MOF structure. A further increase in mass above 600 °C could be due to the formation of bismuth oxide. Bismuth-containing materials may undergo oxidation when exposed to air (an oxygen-rich environment) leading to the formation of bismuth oxides. This can increase the mass as oxygen from the environment is absorbed into the material. The TGA curve for Ag/Bi-MOF follows a similar trend but with some differences due to the presence of silver. The presence of silver might influence the decomposition temperature and residual mass, with a possible higher thermal stability and a different decomposition pathway. The DSC curve for Bi-MOF shows endothermic peaks corresponding to the weight loss events in the TGA curve. An endothermic peak around 100 °C and 400–600 °C indicates the evaporation of solvents and the decomposition of the ligands. Exothermic peaks at higher temperatures could indicate further decomposition and possible crystallization of Bi_2_O_3_. The DSC curve for Ag/Bi-MOF shows similar thermal events with some variations. The presence of silver might lead to additional or shifted exothermic peaks due to interactions between silver and the framework during decomposition. The presence of silver in Ag/Bi-MOF might enhance the thermal stability, as indicated by possible shifts in decomposition temperatures and residual mass. Differences in the DSC curves, such as additional or shifted peaks, suggest that silver influences the thermal behavior and decomposition pathway of Ag/Bi-MOF.^56^

#### 3.1.5 SEM analysis

**Fig. 3:**
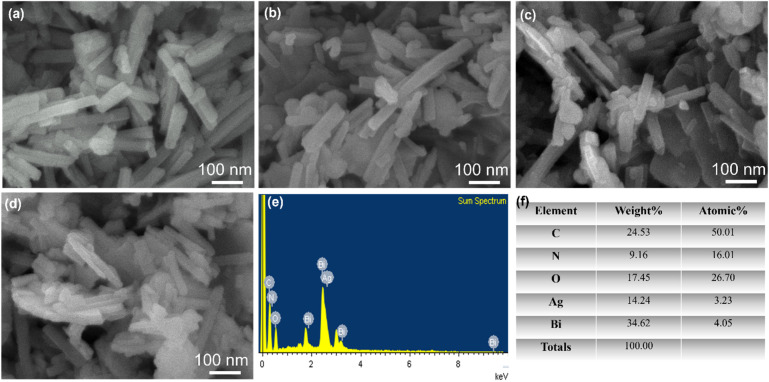
SEM images of (a) Bi-MOF, (b) Bi_2_O_3_, (c) Ag/Bi-MOF, (d) Ag/Bi_2_O_3_, (e) EDX spectrum of the Ag/Bi-MOF sample, (f) elemental weight% and atomic% distribution table of the Ag/Bi-MOF sample.

Scanning Electron Microscopy (SEM) provides detailed images of the surface morphology and particle size of materials (Fig. 3). The SEM image of Bi-MOF shows nanorod shapes that are well-defined and uniformly distributed. The nanorods exhibit smooth surfaces and consistent dimensions, indicating high crystallinity and uniformity in the synthesis process. The nanorods are several micrometers in length and approximately 40 nm in width. Smooth surfaces indicate minimal defects and high-quality crystalline structures. In the case of Bi_2_O_3_, the particles are irregularly shaped and more aggregated compared to Bi-MOF. The morphology is less defined, with significant variation in particle size and shape. Rough and irregular surfaces suggest a less controlled synthesis process and possible variations in material properties. For Ag/Bi-MOF, the structure is similar to Bi-MOF, with well-defined rod-like particles. The surface of the rods appears slightly rougher, possibly due to the incorporation of Ag nanoparticles. There are smaller particles on the surface of the rods, indicating the presence of Ag. The Ag/Bi_2_O_3_ nanorod shows a more defined structure compared to pure Bi_2_O_3_ but still exhibits some aggregation and irregularity. There are small particles present on the surface, indicating the presence of Ag nanoparticles. The surfaces are rough, and the presence of Ag nanoparticles suggests improved material properties compared to pure Bi_2_O_3_.

Fig. 3e is the EDX spectrum of the Ag/Bi-MOF sample, which helps identify the elements present in the sample. The peaks in the spectrum correspond to elements such as carbon, nitrogen, oxygen, silver, and bismuth. Ag and Bi are the main components, with Bi playing a key role in the MOF structure and Ag being added after the modification process. Through the EDX spectrum, the successful synthesis of the Ag/Bi-MOF is confirmed. Fig. 3f is a table analyzing the weight percentage and atomic percentage of the elements in the Ag/Bi-MOF sample. C, N, and O are light elements from the organic structure of the MOF, with weight percentages of 24.53%, 9.16%, and 17.45%, respectively. Ag and Bi are the two main metals, with Bi having the highest weight percentage at 34.62% and atomic percentage at 4.05%. Ag has a weight percentage of 14.24% and an atomic percentage of 3.23%, indicating Ag's supplementary role after the modification process.

### 3.2. The adsorption capacity and photocatalytic degradation analysis

#### 3.2.1 Rhodamine B: bulky molecule structure

**Fig. 4:**
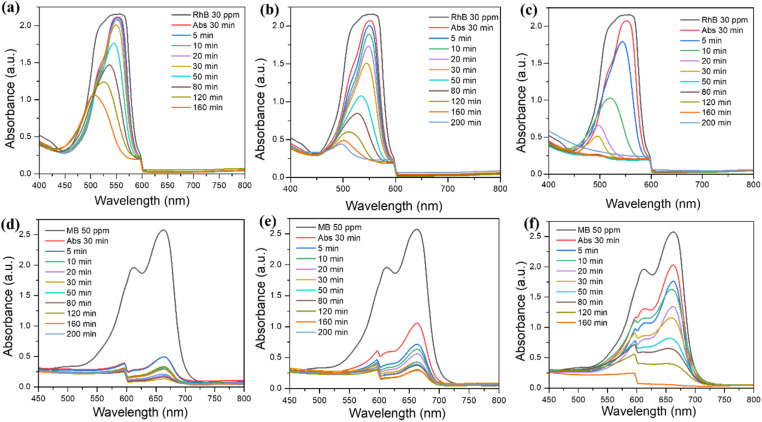
UV-vis absorption and photocatalysis of (a) Bi-MOF, (b) Bi_2_O_3_, and (c) Ag/Bi_2_O_3_ for the removal of Rhodamine B (RhB); UV-vis absorption and photocatalysis of (d) Bi-MOF, (e) Bi_2_O_3_, and (f) Ag/Bi_2_O_3_ for the removal of Methylene Blue (MB).

The comparative analysis of Fig. 4(a–c) provides insightful data on the photocatalytic efficiencies of Bi-MOF, Bi_2_O_3_, and Ag/Bi_2_O_3_ in degrading Rhodamine B (RhB) dye at 553 nm under light irradiation. The results indicate a clear difference in performance among the three materials, primarily influenced by their distinct structural and compositional characteristics.

Fig. 4a demonstrates the performance of Bi-MOF, where the absorbance of RhB is the lowest among the three materials. However, under light irradiation, it still shows catalytic capability and achieves nearly 50% efficiency after 160 minutes. This indicates that Bi-MOF lacks the conditions to adsorb bulky molecules like RhB, which are both hydrophilic and hydrophobic. The likely reason is that the pore structure is not suitable for bulky molecules. Nevertheless, Bi-MOF itself is effective in photocatalytic degradation, likely due to its high surface area, which facilitates the adsorption of RhB molecules onto its surface, bringing them closer to the active sites where photocatalytic reactions occur.

In Fig. 4b, Bi_2_O_3_ shows RhB adsorption but does not significantly improve compared to the case of Bi-MOF. The degradation curves show a faster decrease in RhB concentration over time (up to 200 minutes) compared to Bi-MOF. The improved efficiency may also be due to more effective generation and utilization of reactive oxygen species (ROS) for RhB degradation.^9,57^

The most significant results are seen in Fig. 4c. Ag/Bi_2_O_3_ shows a very small decrease in RhB absorbance, but almost completely degrades the dye within a shorter time. The incorporation of silver nanoparticles into Bi_2_O_3_ substantially enhances the photocatalytic performance. Silver nanoparticles act as electron sinks, improving charge separation and reducing electron–hole recombination, which increases the production of ROS.^58,59^ Moreover, the localized surface plasmon resonance (LSPR) effect of silver nanoparticles under light irradiation amplifies the local electromagnetic field, further boosting photocatalytic activity. This synergistic effect between Ag and Bi_2_O_3_ results in a highly efficient photocatalyst capable of quickly and effectively degrading RhB.

#### 3.2.2 Methylene blue: small molecular structure

Fig. 4d–f provide the UV-vis absorption and photocatalytic degradation of methylene blue using Bi-MOF, Bi_2_O_3_, and Ag/Bi_2_O_3_ as photocatalysts.

In Fig. 4d, the first peak represents the initial concentration of MB at around 662 nm. The second peak shows the MB concentration after 30 minutes of adsorption. The significant decrease indicates that Bi-MOF effectively adsorbs MB. Subsequently, Bi-MOF indicates the gradual degradation of MB by photocatalytic activity. This explains that Bi-MOF, with its high surface area and suitable pore structure, is effective at adsorbing dyes with small and hydrophilic molecular sizes like MB.

Fig. 4e shows MB adsorption on Bi_2_O_3_, which is relatively less effective compared to Bi-MOF, as indicated by a smaller decrease in absorbance. However, the degradation curves display a significant reduction in MB concentration over time, although not as effectively as Bi-MOF. The results suggest that while Bi_2_O_3_ is capable of degrading MB, it has fewer active sites and possibly a lower surface area, which limits its overall adsorption capacity and good catalytic efficiency.

Ag/Bi_2_O_3_ shows the least effective adsorption for MB (Fig. 4f) due to the reduction in pore size from 2.5 to 2.2 nm. However, Ag/Bi_2_O_3_ demonstrates the most substantial decrease in MB photocatalytic degradation over time, achieving near-complete degradation within a shorter period of 160 minutes. The incorporation of silver nanoparticles significantly enhances the photocatalytic performance. Silver nanoparticles act as electron sinks, improving charge separation and reducing electron–hole recombination, which leads to increased ROS production.^60,61^ The localized surface plasmon resonance effect of silver nanoparticles further boosts the photocatalytic activity, making Ag/Bi_2_O_3_ the most efficient photocatalyst.

#### 3.2.3 Influence of Bi-MOF pore structure

##### 3.2.3.1 The feature micropores

The pore structure of bismuth metal–organic frameworks (Bi-MOFs) is a crucial factor that determines their efficiency in photocatalysis applications. Bi-MOFs typically feature micropores with average diameters of 25 nm. This allows for high surface areas and accessible active sites, enhancing adsorption and catalytic properties. Additionally, Bi-MOFs possess high BET surface areas, which indicate their porous nature and high adsorption capacity. This is evidenced by their very high adsorption capacity for MB (80.8%) but limited adsorption for RhB (2.5%) – which has bulky molecules, despite having good physicochemical interaction capabilities with Bi-MOFs.

##### 3.2.3.2 The stability micropores

Bi-MOFs possess high thermal stability and can maintain their structure under high temperature and pressure conditions. Besides, Bi-MOFs can withstand these conditions without significant deformation or structural breakdown. Furthermore, Bi-MOFs are designed to endure high temperatures and boiling organic solvents, enhancing their applicability in harsh environments. This is evidenced by their very effective adsorption capacity, reaching up to 58.9% for Bi_2_O_3_ after being calcined at high temperatures for an extended period. Moreover, after being doped with Ag (Ag/Bi_2_O_3_), the adsorption capacity reaches 21.1%.

**Fig. 5:**
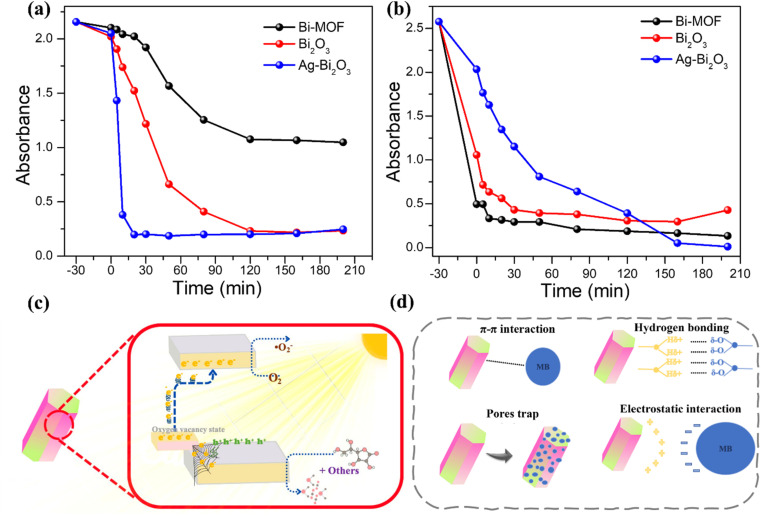
(a) Synthesis, UV-vis absorption, and photocatalysis of Bi-MOF, Bi_2_O_3_, and Ag/Bi_2_O_3_ for the removal of Rhodamine B (RhB); (b) UV-vis absorption and photocatalysis of Bi-MOF, Bi_2_O_3_, and Ag/Bi_2_O_3_ for the removal of Methylene Blue (MB); (c) proposed mechanism of photocatalysis by Bi-MOF derivatives under visible light; (d) four possible adsorption mechanisms of Methylene Blue with Bi-MOF.

The analysis of these figures reveals the impact of material composition on photocatalytic performance. Bi-MOF shows moderate efficiency due to its high surface area and limited pore structure for bulky molecules. However, Bi-MOF still demonstrates weak photocatalytic efficiency. Bi_2_O_3_, on the other hand, has a faster degradation rate due to its metal oxide structure and ROS generation efficiency.^21^ Ag/Bi_2_O_3_ exhibits the highest efficiency, rapidly degrading RhB due to enhanced charge separation, reduced recombination, and the plasmonic effects of silver nanoparticles. These results underscore the importance of optimizing material properties and compositions to develop highly efficient photocatalysts for environmental applications (Fig. 5a).

The results indicate that Ag/Bi_2_O_3_ is the most efficient photocatalyst for degrading Methylene Blue, followed by Bi-MOF and Bi_2_O_3_. The incorporation of silver nanoparticles into Bi_2_O_3_ significantly enhances its photocatalytic properties, making Ag/Bi_2_O_3_ the most promising candidate for environmental remediation applications (Fig. 5b).

Fig. 5c illustrates the photocatalytic mechanism of Bi-MOF derivatives under visible light. Bi-MOF derivatives absorb visible light, exciting electrons from the valence band to the conduction band, creating electron–hole pairs. Excited electrons move to the surface of the Bi-MOF derivatives and react with O_2_, forming reactive oxygen species like ·O_2_^−^. Holes (h^+^) oxidize H_2_O or OH^−^ to generate hydroxyl radicals (·OH), which are highly oxidative. These reactive species (·O_2_^−^ and ·OH) oxidize organic pollutants such as Rhodamine B and Methylene Blue, degrading them into non-toxic products. Additionally, other factors support the photocatalytic process of Bi-MOF and its derivatives: (i) oxygen vacancy state: which increases the formation of reactive oxygen species; (ii) π–π interactions: enhance adsorption and degradation of organic molecules; (iii) hydrogen bonding and electrostatic interactions: aid in stabilizing and reacting with pollutants.

Rhodamine B and Methylene Blue are cationic dyes that carry a positive charge in solution at neutral pH (around pH 7). At neutral pH, the zeta potential of RhB reaches a value of +22.4 mV. However, the zeta potential of MB reaches a higher value of +34.3 mV. On the other hand, Bi-MOF is a negatively charged adsorbent (−12.71 mV), which can interact with the positively charged MB/RhB through electrostatic interactions (Fig. 5d). However, when Bi-MOF is calcined, it is converted to Bi_2_O_3_, which has a lower surface area, consequently leading to a decrease in the adsorption capacity of the Bi_2_O_3_ nanorods. The interactions between MB and the metal framework of Bi-MOF can induce the trapping of MB molecules in the pore structure of Bi-MOF, improving the adsorption capacity. However, for bulky molecules like RhB, the adsorption is negligible due to the limitations of the pore structure of Bi-MOF. Besides, some π–π interactions of MB with the benzene ring structure might explain the adsorption mechanism of MB in the Bi-MOF. However, the π–π interactions disappeared in the Bi_2_O_3_ sample because the benzene rings of Bi_2_O_3_ are destroyed, leading to poor adsorption capacity. The hydrogen bonding interactions were observed between Bi-MOF and MB. These interactions highlight the potential application of Bi-MOF-based derivatives for removing hazardous organics in water,^62^ based on electrostatic interactions, hydrogen bonding, π–π stacking interactions, and pore/size-selective adsorption Table 1.

**Table 1:** Comparative table of photodegradation of rhodamine B (30 ppm) and methylene blue (50 ppm) using Bi-MOF, Bi_2_O_3_, and Ag/Bi_2_O_3_

Material	RhB/MB	Degradation performance after 50 min	Maximum degradation efficiency	Time for maximum degradation efficiency
Bi-MOF	RhB	27.32%	51.44%	200 min
	MB	88.66%	94.80%	200 min
Bi_2_O_3_	RhB	69.29%	89.89%	160 min
	MB	84.70%	88.55%	160 min
Ag/Bi_2_O_3_	RhB	91.33%	91.33%	50 min
	MB	68.59%	99.61%	200 min

**Fig. 6:**
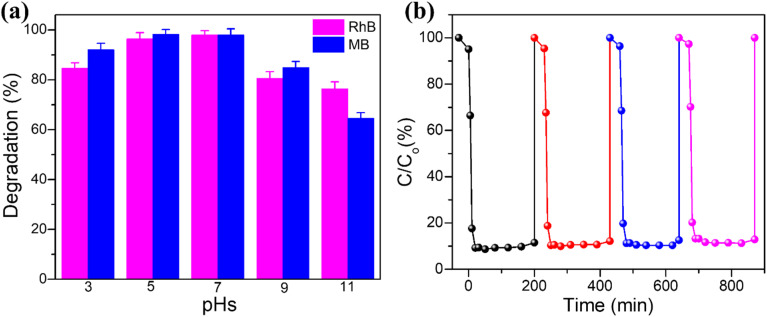
(a) The influence of different pH values (3, 5, 7, 9, 11) on the degradation efficiency of RhB and MB by Ag/Bi_2_O_3_; (b) recycling performance of RhB degradation by Ag/Bi_2_O_3_ after 4 cycles.

Fig. 6a in the provided image shows the effect of different pH values (3, 5, 7, 9, 11) on the degradation efficiency of two dyes, Rhodamine B (RhB) and Methylene Blue (MB), using Ag–Bi_2_O_3_. At low pH (pH = 3), degradation still occurs with high efficiency. Specifically, the degradation efficiency of RhB is close to 84.6%, while MB reaches 92.1%. At a higher pH (pH = 5), the degradation efficiency of MB increases significantly to 96.5%, and RhB increases to its highest value, reaching 98.3%. At neutral pH (pH = 7), the degradation efficiency for both dyes reaches a very high value, around 98% for both RhB and MB. However, the degradation efficiency decreases, reaching around 80.6% for RhB and 85% for MB, when tested at alkaline pH (pH = 9). At strong alkaline pH (pH = 11), the degradation efficiency is the lowest, reaching 76.5% for RhB and 64.6% for MB.

Ag/Bi_2_O_3_ achieves effective degradation of both RhB and MB across a wide pH range from 3 to 11. The degradation efficiency of MB is higher and effective across a broader pH range (3–9), with particularly high degradation efficiency in acidic environments, specifically at pH 5. For RhB, the optimal condition for high degradation efficiency is in a neutral environment (pH = 7). Under strongly alkaline conditions (pH = 11), the degradation of RhB is still better compared to MB. These results can be used to determine the optimal pH conditions for degrading different dyes with Ag–Bi_2_O_3_, aiding in optimizing waste dye treatment processes for practical applications.

The reusability of Ag/Bi_2_O_3_ was assessed through recycling experiments. The used adsorbent was centrifuged and soaked in 100 mL of methanol for desorption under ultrasonication for 90 minutes. The recovered Ag/Bi_2_O_3_ was then washed three times with methanol and dried at 60 °C for the next adsorption cycle (Fig. 6b). The degradation efficiency remained high, with the concentration quickly dropping to nearly 10% within a similar timeframe as the first cycle. Although there was a slight reduction in efficiency, the concentration still notably decreased within the same period. The Ag/Bi_2_O_3_ catalyst consistently and effectively degraded RhB over four cycles, with only a minor decrease in efficiency in the later cycles. Initially, the catalyst exhibited high degradation efficiency, reducing the RhB concentration to nearly 10% within the first few minutes of each cycle. Despite a slight drop in efficiency after multiple cycles, likely due to the weight loss of ZIF-8 during the recycling process, the catalyst maintained significant degradation capability. These findings suggest that Ag/Bi_2_O_3_ can be reused multiple times for RhB degradation with minimal performance loss, highlighting its potential for sustainable and cost-effective dye degradation and wastewater treatment applications.

**Fig. 7:**
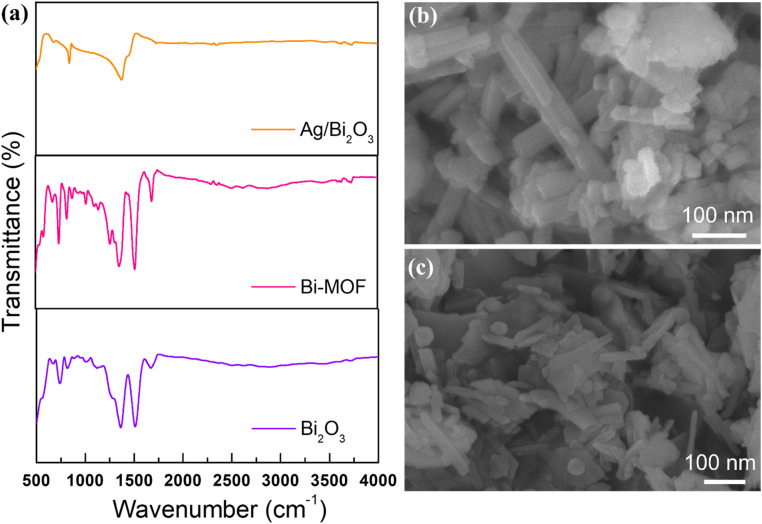
(a) FTIR spectra of Bi-MOF, Bi_2_O_3_, and Ag/Bi_2_O_3_ after the adsorption and photocatalysis process, (b) SEM images of Bi-MOF and (c) Ag/Bi_2_O_3_ after the photocatalysis process.

In the case of Bi-MOF, the fundamental peaks are retained compared to the photocatalytic process. Specifically, the strong intensity peaks in the range of 1254 and 1377 cm^−1^ represent C–O bonds in carboxylate groups and Bi–O bonds. Additionally, the peaks in the 742 and 814 cm^−1^ region contain a series of peaks unique to the specific Bi-MOF structure. After the photocatalytic process, the FTIR spectrum of Ag/Bi_2_O_3_ still retains the fundamental peaks at 1384 cm^−1^, along with the peak at 840 cm^−1^ (Fig. 7a). The comparison of the FTIR spectra before and after the catalytic process for Bi-MOF, Bi_2_O_3_, and Ag/Bi_2_O_3_ shows characteristic peaks for functional groups in the structure. Bi-MOF, Bi_2_O_3_, and Ag/Bi_2_O_3_ all exhibit characteristic peaks for functional groups within their structure. After the photocatalytic process, the peaks related to metal–oxygen bonds (Bi–O and Ag–O) remain unchanged, despite the strong impact of the photocatalysis on the organic structure, which had little effect on the metal bonds. Additionally, some absorption bands weaken or shift slightly, indicating minor changes in chemical bonding. This confirms the involvement of the materials in the photocatalytic reaction.

The SEM images of Bi-MOF and Ag/Bi_2_O_3_ show that the bismuth nanorod structure remains largely unchanged. In the case of Ag/Bi_2_O_3_, Ag particles are still observed attached to the nanorods. This demonstrates the structural stability of bismuth MOF and its derivatives after the catalytic and adsorption processes (Fig. 7b).

## 4. Conclusions

This study successfully synthesized and characterized bismuth metal–organic framework nanorods and their derivatives, such as Ag/Bi_2_O_3_. The Bi-MOF nanorods exhibited significant photocatalytic activity under visible light, particularly with the addition of silver (Ag), which enhanced charge separation efficiency and reduced electron–hole recombination. This led to improved photocatalytic performance, especially in the degradation of organic pollutants like Rhodamine B and Methylene Blue. The results demonstrated that Bi-MOFs and their derivatives possess excellent chemical stability and high efficiency in photocatalytic applications, even when exposed to high temperatures and a wide pH range. Their large surface area and microporous structure facilitate the selective adsorption of small organic molecules like MB. The pores and large surface area not only provide numerous active sites but also enhance the interaction between reactants and the catalyst surface, thereby improving photocatalytic efficiency. On the other hand, these pores can selectively adsorb small molecules like MB, significantly impacting the adsorption process. Bi-MOFs and their derivatives operate optimally across a broad pH range, from acidic to alkaline environments, where strong oxidizing hydroxyl radicals (·OH) are readily formed, aiding in the effective degradation of organic compounds. The study also showed that Bi-MOFs and their derivatives can be reused multiple times without significant loss in performance. This reusability is crucial for practical applications, indicating the potential of Bi-MOFs and their derivatives for sustainable and efficient environmental remediation applications.

## Data availability

Data will be made available upon request.

## Conflicts of interest

The authors declare that they have no known competing financial interests or personal relationships that could have appeared to influence the work reported in this paper.
